# A colonial-nesting seabird shows limited heart rate responses to natural variation in threats of polar bears

**DOI:** 10.1098/rsos.221108

**Published:** 2023-10-04

**Authors:** Erica A. Geldart, Oliver P. Love, Andrew F. Barnas, Christopher M. Harris, H. Grant Gilchrist, Christina A. D. Semeniuk

**Affiliations:** ^1^ Great Lakes Institute for Environmental Research, University of Windsor, Windsor, Ontario, Canada; ^2^ Department of Integrative Biology, University of Windsor, Windsor, Ontario, Canada; ^3^ National Wildlife Research Center, Environment and Climate Change Canada, Ottawa, Ontario, Canada; ^4^ School of Environmental Studies, University of Victoria, Victoria, British Columbia, Canada

**Keywords:** common eider, polar bear, heart rate response, Arctic colonial nesting seabird, predation threat, dynamic risk assessment

## Abstract

Several predator–prey systems are in flux as an indirect result of climate change. In the Arctic, earlier sea-ice loss is driving polar bears (*Ursus maritimus*) onto land when many colonial nesting seabirds are breeding. The result is a higher threat of nest predation for birds with potential limited ability to respond. We quantified heart rate change in a large common eider (*Somateria mollissima*) breeding colony in the Canadian Arctic to explore their adaptive capacity to keep pace with the increasing risk of egg predation by polar bears. Eiders displayed on average higher heart rates from baseline when polar bears were within their field of view. Moreover, eiders were insensitive to variation in the distance bears were to their nests, but exhibited mild bradycardia (lowered heart rate) the longer the eider was exposed to the bear given the hen's visibility. Results indicate that a limited ability to assess the risks posed by polar bears may result in long-term fitness consequences for eiders from the increasing frequency in interactions with this predator.

## 1. Introduction

Rapid environmental change is impacting inter-specific relationships across multiple ecosystems worldwide [1]. Specifically, changes in phenology of seasonal life-history events [2,3] or distribution of species [3] can create new species relationships or exacerbate existing interactions between antagonistic species, such as between predator and prey [4]. What is unclear is the extent to which such prey have the necessary evolved mechanisms to recognize and respond to this degree of change in predator regime [5]. A prey's experience with a predator on both evolutionary (i.e. experience over multiple prey generations) and ecological timescales (i.e. recent experience over a prey's lifetime) influences their ability to detect and respond adaptively to the threat posed by the new/historically rare predator*.* Characterizing whether prey can appropriately assess the risks posed by a predator with which they have limited eco-evolutionary experience can provide insight into whether prey can mitigate the increased frequency of predator interactions expected by ecosystem change [6].

An individual's ability to respond adaptively to a predation threat depends, in part, on their capacity to recognize the level of predation risk the predator poses and adjust their responses accordingly [7]. For instance, a predator's physical (e.g. size) and behavioural (e.g. speed, angle of approach and gaze direction) characteristics can provide information on their likely future action [8–10]. However, these more subtle cues are not always easily observable. Instead, in the context of more coarse-scale predator behavioural information (e.g. presence–absence or location), an individual's perception of risk may be influenced by the distance from and duration of exposure to the predator (e.g. [7,11]). In the wild, individuals typically use distance as a measure of predatory imminence, and regulate their trait responses based on this perceived likelihood of detection and capture by the predator [12]. When a threshold distance is passed by an approaching predator, the perceived risk of predation grows, and prey will switch to a fight-or-flight response that can generate higher vigilance, blood circulation to gross muscles and accelerated heart rate [13]. An individual's response to the threat of predation can furthermore be influenced by the amount of time prey are in the presence of a predator. For example, exposure duration of a threat stimulus influences the stress response (e.g. [11,14]), where a higher duration of interaction is associated with a greater probability of detection and capture. The magnitude of this effect will be further impacted by the availability of visual information to prey [15]. For instance, the range at which visual cues are likely to be informative and used by prey to assess threats can be influenced by the spatial structure of the environment such as topography and cover. Any information deficit resulting from a small spatial-visibility range will also play a role in risk perception and should affect prey responsiveness.

Assessing an individual's perception of predation risk via behavioural markers is easily accomplished by evaluating antipredator responses such as vigilance levels and escape responses [12,16–20]. However, in some cases prey may display a physiological response to threat without exhibiting obvious behavioural ones [21]. In addition, individuals can exhibit signs typically associated with antipredator behaviour in the absence of threat (e.g. seemingly vigilant individuals are actually searching for food [17]). Physiological measures, such as the release of stress hormones and changes in heart rate, can provide an alternative objective evaluation of fear in animals that underlies an individual's level of perceived predation risk [11,22]. Modulation of heart rate is considered to be an immediate fear response to the detection of acute predation risk: both sympathetic (acceleratory influence) and parasympathetic (deceleratory influence) branches of the autonomic nervous system that mediate heart rate are activated within seconds following the perception of a threat [12,23]. Magnitude of change in heart rate from baseline levels can therefore be used as a metric to assess perceived level of predation threat across variation in realized predation risk.

Here we assess the perceived risk of polar bears (*Ursus maritimus*) by incubating female common eiders (*Somateria mollissima*, hereafter ‘eider’) on an island breeding colony in Nunavut, Canada. We used non-invasive methods to measure eider physiological responses and bear activity. Over the past few decades, sea-ice break-up has been advancing at a more rapid pace than climate-induced advancement in bird nest initiation dates and hatching (e.g. common eider, thick-billed murre *Uria lomvia*, and glaucous gull *Larus hyperboreus* [24–26]). Resultantly, increasing spatial and temporal overlap is occurring between incubating colonial-nesting seabirds and polar bears on land. As a consequence of this recent phenological match, polar bear egg predation in Arctic breeding seabird colonies has steadily and rapidly increased [27]*.* Eiders have evolved several adaptations to reduce predation by a land-based mammalian egg predator (Arctic fox, *Vulpes lagopus*) and egg scavengers (*Larus* gulls). In the Arctic, eiders commonly nest on islands inaccessible to foxes [28] and form high-density nesting aggregations for communal defence and predator warning [29,30]. These nesting strategies are not effective against predation by polar bears that can swim to islands [31]; and bears are actually more likely to visit and remain on larger eider nesting colonies [24]. These increased encounter rates are now associated with reduced nest success in some eider colonies, and this impact has exceeded that caused by their traditional egg predators at some locations [24,26]. Whether eiders recognize the fitness risks posed by polar bears is unknown. Combining remotely deployed heart-rate monitoring systems with an array of trail cameras that simultaneously monitored polar bear activity, we quantified eider heart rate as a metric for perceived predation threat in the presence of bears. Several other studies have successfully used heart rate as a metric for perceived risk by eiders. In a recent study, we approached eiders with different predator imagery (polar bear, Arctic fox and control), and birds displayed a higher magnitude of response to the fox than bear compared with the control, but exhibited a fear bradycardia response leading up to flushing regardless of predator stimulus [32]. This fear bradycardia response was also observed for three nesting eiders encountering actual approaching polar bears in the same study (although this response was never compared with an eider's heart rate in the absence of a polar bear). Eiders have also been shown to display tachycardia during researcher handling [33]. Owing to eiders' rapidly changing ecological experience with polar bear egg predators, we hypothesized that eiders do not dynamically update their assessment of risk. As such, we predicted that eiders would exhibit a heart rate response different from baseline in the presence of a polar bear, but, that eider heart rates would be insensitive to both the distances to bears, and the ratio of risk between predator exposure (i.e. the amount of time a bear remained visible) and the area of the eider's viewshed.

## 2. Material and methods

### 2.1. Study species and area

We conducted work on East Bay (Mitivik) Island (EBI), Nunavut, Canada (64°02′ N, 81°47′ W), a small (24 ha), low-lying (less than 8 m elevation change) eider nesting colony within Southampton Island's Qaqsauqtuuq (East Bay) Migratory Bird Sanctuary (electronic supplementary material, figure S1). The island experiences almost continuous daylight during the nesting season in June and July [34], and the landscape is flat and composed of patches of low-lying tundra vegetation, granite rocks and several small ponds. The island hosts the largest known common eider breeding colony in the Canadian Arctic (Inuit Nunangat) [35]. Eiders on EBI initiate laying in late June/early July [36], and undergo an approximate 24-day incubation period where they do not feed [37]*.* According to local Inuit members of the Hunters and Trappers Association of the nearby community of Salliq (Coral Harbour), Nunavut, the surrounding East Bay area is also an important migratory route for the Foxe Basin polar bear subpopulation in transit towards summer's last land-fast ice in northern Foxe Basin. However, in recent years, earlier spring sea-ice break-up in northern Hudson Bay and reduced access to seal prey have driven polar bears ashore to EBI earlier, where bears now overlap with eiders during laying and incubation [24]. As a result, eiders are experiencing increasing nest predation pressure by polar bears [38–40], insofar that duckling recruitment rates have been documented close to zero in more recent years (H.G.G. and O.P.L. 2019, personal observations).

### 2.2. Heart rate monitoring

We recorded the heart rate of incubating eider hens using an artificial-egg heart-rate monitor added to the clutch. Artificial-egg heart-rate monitors have been used previously on other bird species throughout the length of their incubation and shown to be minimally invasive [41]*.* Full details on the construction and deployment of heart-rate monitors are outlined in Geldart *et al*. [32]. Briefly, heart-rate monitors had Electret condenser microphones (PUI Audio model AOM-5024L-HD-R) embedded in a three-dimensionally printed eider egg. Each artificial egg was wired to a digital audio recorder (Tascam DR-05X) in a weatherproof, camouflaged box. We constructed artificial eggs so the side of the egg with microphones continuously faced upwards against the eider's brood patch.

We deployed heart-rate monitors in active eider nests (*n* = 11) on 24, 25 and 28 June 2019. Nests contained one to five eggs at visitation (i.e. clutch size during laying or incubation). We deployed heart-rate monitors in areas of low nesting densities to limit researcher-induced disturbance within denser portions of the colony. GPS location of study nests confirmed they were located 302 ± 162 m (mean ± s.d.; range: 11–560 m) apart from each other (electronic supplementary material, figure S1). The devices collected continuous audio recordings of the incubating eiders' heart rate for 11–12 days. The storage box was placed approximately 1 m away from the nest, and both the wire and box were secured and concealed with the surrounding terrain. Birds returned to their nest on average within 1.19 h (0.00139–6.61 h range) after equipment was deployed in their nest. We observed no predation at these nests while the female was away due to researcher activities. To avoid disruption to the colony, we retrieved all heart-rate equipment within 2–3 h on 19 July after clutches had hatched or been predated.

### 2.3. Polar bear locations in relation to eiders

We deployed a large array (*n* = 84) of GPS-marked remote trail cameras (Browning Inc. model: BTC-5HDPX) across the island (electronic supplementary material, figure S1) to estimate polar bear locations in relation to focal eiders. We mounted a subset of the trail cameras (*n* = 35, set to motion-triggered 30 s videos, equipped with a 128GB SD card) approximately 6 feet above ground level (AGL) on wooden posts or fixed bird observation blinds (electronic supplementary material, figure S2, deployed on 11–14 and 28 June); and we deployed the remaining cameras (*n* = 49, set to 2 min videos once triggered, equipped with a 256 GB SD card) at ground level approximately 1 m from active eider nests (electronic supplementary material, figure S2, deployed on 18, 24, 25 and 28 June 2019). See electronic supplementary material, table S1 for additional camera settings used. At time of deployment, we powered the camera, set date and time accordingly, and recorded the camera's GPS location and cardinal direction. All cameras collected data from the time of their deployment up to 19 July at the latest (i.e. collected data for 22–39 days (mounted cameras) and 2–26 days (ground-based cameras), start and end date inclusive). A subset of the ground-based trail cameras (*n* = 11, electronic supplementary material, figure S1) were paired with the heart-rate monitored nests.

**Figure 1.**
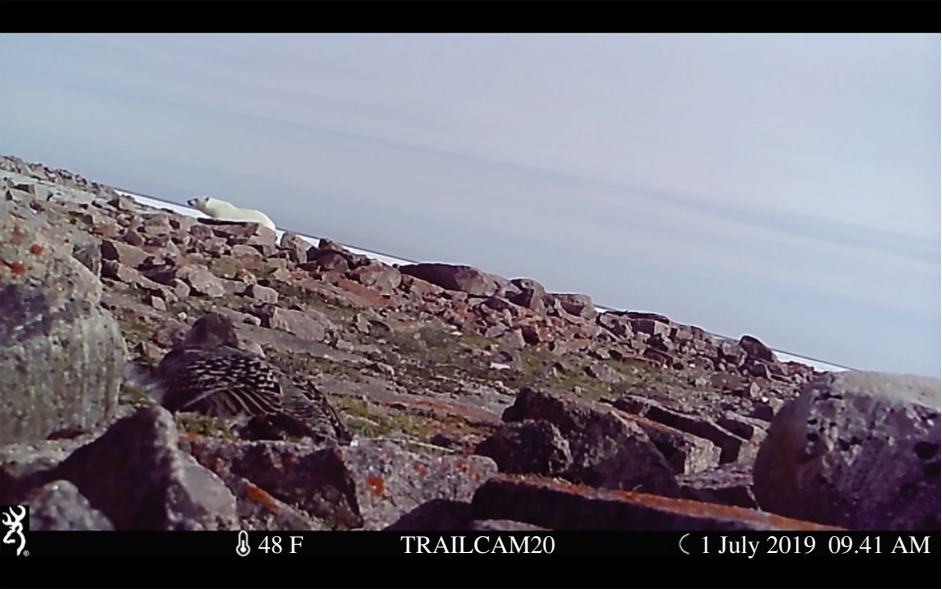
Photo of trail camera (Browning inc. model: BTC-5HDPX) footage showing a focal incubating female common eider (*Somateria mollissima*) and polar bear (*Ursus maritimus*) on East Bay Island, Nunavut, Canada at 9.41 on 1 July 2019.

For safety purposes, polar bear presence on EBI was discouraged by the research team during the daytime from late May to 29 June, and according to the trail camera data, no bears were present on the island during this time while the heart-rate monitors were collecting data. However, researchers intentionally left the island between 30 June and 19 July, allowing bears to forage freely. To validate the ability of our camera array to detect bears when they were present on the island, we constructed a spatially explicit agent-based simulation model of bears moving through a subset of our camera array (*n* = 35) using manufacturer-reported detection distances and angles. We found a high probability that bears were detected (84.8% across 1000 model runs) using only 35 cameras, and are therefore confident our full array of 84 cameras was able to detect bears given their true presence on the island. For full details on these simulations, see electronic supplementary material. To identify trail-camera footage with bear(s) present, two observers (E.A.G. and C. Simone) reviewed all the videos from the 35 mounted trail cameras, and one observer (E.A.G.) reviewed footage from the 49 ground-based trail cameras (figure 1). All videos (*n* = 293) from 24 June to 10 July (i.e. range of eider heart-rate monitoring period) with polar bear(s) present were used to locate bears on a georeferenced, true colour, 3 cm ground sampling distance (GSD) orthomosaic map of EBI produced via aerial photography in late June 2019 when the island was free of snow. We used ArcMap v. 10.6.1 (Esri Inc, Redlands, CA; [42]) to plot locations of bears on the island mosaic. A single observer (E.A.G.) matched rock patterns, vegetation outcrops and other landscape features such as research infrastructure and pond edges between the trail camera videos and the island mosaic. We marked bear locations at 15 s intervals in the videos (i.e. at video timestamp 0.00, 0.15, 0.30, etc.) on the island mosaic using the *Point* tool (electronic supplementary material, figure S3). We created buffers of 1, 5 or 10 m radiuses around each bear location point using the *Buffer* tool, based on the observer's level of certainty to accurately define the bear's exact location on the island mosaic to acquire a conservative estimate of bear location. Only 3.57% of our samples had bear location points with 10 m buffers, whereas 28.57% and 67.86% had location points associated with 5 and 1 m buffers, respectively. This reflects the high resolution of the island photography itself which greatly helped us identify key features of specific locations. Importantly, all bear location points involved in our closest-to-nest location points had associated buffers within 5 and 1 m (the majority) buffers. When multiple bears were detected on the island simultaneously, bear IDs were established based on their different morphometric features that differentiated them such as body size, scars, wounds or markings on their fur. Despite the presence of multiple bears, there were no instances of eiders being exposed to more than one bear at the same time (see *Estimating bear exposure within eider viewsheds* below).

### 2.4. Estimating bear exposure within eider viewsheds

To calculate the amount of time polar bears were within viewing distance to an eider, we first estimated the area of a focal eider's viewshed given the island's topography [15]. We performed a viewshed analysis for each focal eider nest location by first converting the island orthomosaic map into a digital surface model (DSM) to produce a raster grid, and then using ArcMap's *Viewshed* tool. We estimated viewshed for each eider by including a 14 cm vertical offset to account for the approximate eye level of nesting eiders (i.e. observer point; measured using a sitting model eider) and a 1.5 m offset to account for the approximate average height of a polar bear standing on all fours (i.e. the *z*-value being considered for visibility) [43]. We then used the *Raster to Polygon* tool to convert each viewshed raster to a polygon layer. We manually clipped out a polygon of EBI using the *Polygon* tool, and we used ArcMap's *Clip geoprocessing* tool to clip each of our viewshed polygons (i.e. input feature) within our island polygon (i.e. clip feature). Finally, we extracted the area (in m^2^) of the clipped polygon layers, representing the area that each focal eider could visually detect a polar bear on EBI.

### 2.5. Heart rate quantification

A single researcher (E.A.G.) reviewed eider heart-rate recordings using the sound analysis software Audacity v. 2.3.2 [44]. We synchronized heart-rate monitors and trail cameras used to define bear locations (see above) to the nearest minute from the clock of an iPad (Apple Inc.), which could have created a maximum 120 s clock offset between the two devices. To quantify eider heart rate in response to bears, while accommodating for any differences in temporal resolution between the trail cameras and the heart-rate monitors, we collected a 120 s sample (i.e. further broken down into 12, 10 s intervals) around the midpoint of a period when a bear was observed within an eider's viewshed for a series of consecutive minutes. Multiple heart-rate samples were collected within a bear event whenever possible and spaced at least 1 min apart. We considered bear events (periods of time when a bear was observed within an eider's viewshed) separate and distinct if they were separated by gaps of more than 5 min. We estimated baseline, resting heart rate by collecting heart-rate samples at least 30 min (maximum 58 min) before each bear event. This sampling method allowed us to simultaneously account for any diel variation in heart rate caused by labile biotic and abiotic conditions. In one instance, two bear events occurred within 30 min of each other, so they received the same baseline sample taken 30 min before the first event. Additionally, to ensure baseline heart rates were collected during a predator-free period, these samples were verified that a bear had been last observed within a hen's viewshed a minimum of 55 min prior, as confirmed through trail cameras. Within this heart-rate sampling period, we similarly collected a 120 s sample (i.e. 12, 10 s intervals) of ‘clean’ baseline heart rate from each eider to calculate an average baseline for each female for calculating heart-rate change (see details below). The opportunistic nature of our sampling method generated different sample sizes for the number of bear events and the number of heart-rate samples associated with each of them. We extracted all of the sample intervals as .wav files, and heartbeats were counted aurally at least twice to avoid measurement error. We excluded sample intervals from analysis for which the sound quality was too poor to allow accurate quantification of heart rates. No researchers were present on the island when heart-rate samples were collected.

To examine drivers of heart rate variation, we calculated the magnitude of change in eider heart rate response to polar bears from baseline (as beats/10 s), hereafter termed ‘ΔHR’ (equation (2.1)). In equation (2.1), HR_baseline_ is the average baseline heart rate for a female from each sample interval collected prior to each bear event. In cases when there were multiple samples taken within a prolonged bear event, or bear events occurred less than an hour apart for an individual eider, a single baseline sample was paired with these multiple respective bear-induced samples. A positive ΔHR therefore represents an increase in heart rate from baseline during a bear event, whereas a negative value indicates a decrease in heart rate.

2.1 $$\Delta \mathrm{HR}={\mathrm{HR}}_{\mathrm{bear}}\text{–}{\mathrm{HR}}_{\mathrm{baseline}}.$$

### 2.6. Estimating eider distance and ratio of exposure risk to bears

We estimated the distance (in m) between a nesting focal eider and polar bears observed within their viewshed using ArcMap's *Measure* tool. We collected distance measurements for all bear observation points included in each heart-rate sampling period, and calculated the average distance for each heart-rate sample. We estimated the exposure duration of individual eiders to bears by calculating the duration of a bear event within the eider's viewshed (in min), and we corrected exposure duration to the bear for the size of viewshed (in m^2^) to estimate a ratio of exposure risk (min m^−2^). We then assigned these ratios to any heart-rate samples collected within their respective bear event.

### 2.7. Eider incubation stage

To account for additional covariates that could influence heart rate, we quantified incubation stage for each focal hen (i.e. stage of embryo development). During deployment of the artificial egg, the first-laid egg (i.e. egg that was darkest in colour) from each study nest was collected and immediately candled [45]. This method provided estimates of the number of days the first-laid egg had been incubated on the equipment-deployment day allowing us to estimate the incubation stage for focal hens on the day of specific bear events.

### 2.8. Air temperature and wind speed

To examine meteorological conditions for each focal hen during heart-rate sampling to polar bear presence, we collected measurements of air temperature and wind speed from weather meters (Kestrel 5500) deployed at multiple locations within the colony. Weather meters recorded parameters every 30 min. For each eider, we sampled weather from the meter that was nearest to the focal nest (mean ± s.d.: 86 ± 40 m, range: 28–135 m) and we sampled these measurements at the closest 30 min interval to our heart-rate sampling times to bears.

### 2.9. Statistical analyses

To first determine whether eider hens were responsive to the risk of predation, we used a linear mixed model (LMM) using the *lmer4* package [46] on paired samples to compare heart rates recorded during the baseline period versus in the presence of a polar bear. For this model, we included a nested random effect structure for heart-rate sample number, nested within bear event, nested within eider ID, and a separate term for bear event nested within eider ID.

We used an LMM to analyse eider ΔHR to polar bears. Fixed effects included distance to the bear (m), ratio of exposure risk to the bear (min m^−2^), incubation stage (days), air temperature (°C), wind speed (m s^−1^) and the interaction between the distance from the focal eider to the polar bear and the ratio of exposure risk. We included the same random effect structure as described above. We tested fixed effects using a backward elimination procedure by fitting the full model for ΔHR with maximum-likelihood (ML) estimation [47]. Main effects and the interaction term were tested sequentially using the backward elimination procedure, leaving only the fixed effects associated with the outcome in the final model, considering a 10% level of significance (i.e. a standard retention criterion for backward elimination, [48]). The final most competitive model with retained fixed effects was refitted using restricted maximum likelihood (REML) estimation. We assessed the variance explained by the fixed effects (i.e. marginal *R*^2^) and the variance explained by the entire model, including both fixed and random effects (i.e. conditional *R*^2^) for our model, using the package *MuMIn* [49]. We assessed model fit by comparison of second-order Akaike information criterion (AICc) scores between all competing models in the backward elimination process, as well as an intercept-only model, all fit with ML estimation [47], using the package *MuMIn* [49].

To aid in interpreting our ΔHR findings, we used an LMM to test whether prolonged exposure to a bear had an effect on heart rate, by modelling eider heart rate in the presence of a bear as a function of sample interval within a bear event (ordinal categorical: 1–36; i.e. time into a bear event). Note that the greater value of sample interval is associated with a longer time that a bear spent in their viewshed. For this model, we included a nested random effect structure for bear event nested within eider ID. Any effect on our measures of eider heart rate was determined with pairwise comparisons using Tukey's honestly significant difference (HSD) in the *lsmeans* package [50].

We performed analyses in R v. 3.5.0 [51], with an RStudio interface [52]. All data manipulation was done with packages *dplyr* [53] and *tidyverse* [54]. We checked variance inflation factors (VIF) (i.e. multi-collinearity) for all predictor variables using the *car* package [55]; and model residuals were assessed by visual inspection of Q–Q plots. Because the distribution of ΔHR was right skewed, these data were log transformed (equation (2.2)). For all statistical significance tests, we used *α* = 0.1. All plots of predicted response variables, calculated under the package *ggeffects* [56], were constructed under the package *ggplot2* [57].

2.2 $$\log\;\Delta \mathrm{HR}=\log_{10}(\Delta \mathrm{HR}+10).$$

## 3. Results

We collected 20 baseline heart-rate samples (consisting of 237, 10 s sample intervals), and 28 heart-rate samples when polar bears were present (consisting of 276, 10 s sample intervals), from eight incubating eider hens that would have observed 21 polar bear events from their nests (30 June–6 July). This dataset was used in all subsequent analyses. We estimated that 10 individual bears were involved in our bear events who ranged in age from subadult to adult. Bears were in average to good body condition with none observed to be emaciated nor obese [58], so we do not expect variation in eider responses to be caused by differences in physical characteristics of the bears (e.g. size) [15]. Only one bear event led to nest predation (i.e. at least one egg eaten) for one of our study hens. All other successful predation attempts occurred outside of our sampling periods. We were able to collect between one and seven (mean = 3.5) heart-rate samples from eight individual eiders during the presence of a bear. We were unable to collect heart rate from the other three study hens because they either did not have a bear present in their field of view during the heart rate monitoring, the bird was not on her nest when a bear was present, or we were unable to collect a sample of sufficient audio quality.

**Figure 2.**
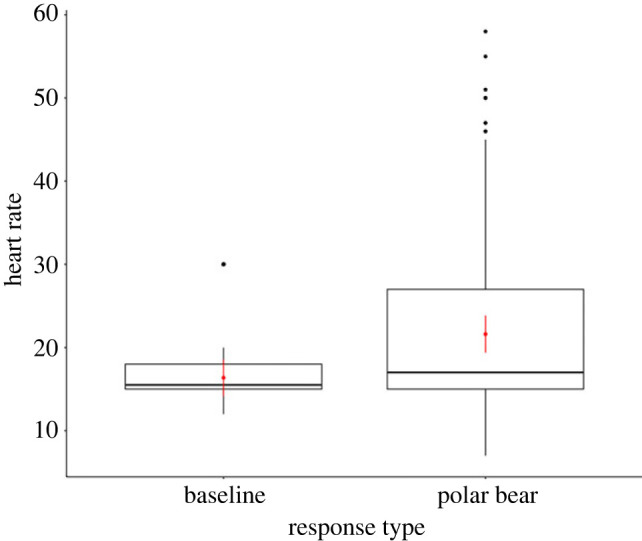
Comparison of baseline heart rate (beats/10 s) versus heart rate in the presence of a polar bear (*Ursus maritimus*) in incubating female common eider (*Somateria mollissima*) on East Bay Island, Nunavut, Canada. Red points correspond to model predicted estimates of heart rate at both baseline and during bear presence, and vertical lines are their associated 95% confidence intervals.

Baseline heart rate ranged from 12 to 30 beats/10 s (mean = 16.4 beats/10 s) and heart rate in the presence of a polar bear ranged from 7 to 58 beats/10 s (mean = 21.4 beats/10 s). Heart rate in the presence of a polar bear was significantly higher than baseline heart rate (Tukey's HSD: *p* < 0.0001, estimate ± s.e. = 5.26 ± 0.44; figure 2 and table 1). Random effects accounted for most of the variance explained by our model (marginal *R*^2^ = 0.11, conditional *R*^2^ = 0.52, table 2).

**Table 1.** Model parameter estimates and standard error for fixed effects used to explain variation in heart rate (baseline versus bear presence) among nesting female common eiders (*Somateria mollissima*) on East Bay Island, Nunavut, Canada.

model parameter	estimate ± s.e.
intercept	16.364 ± 1.130
heart rate to bear presence^a^	5.256 ± 0.442

^a^Reference category = baseline heart rate.

**Table 2.** Model parameter variance and standard deviation for random effects used to explain variation in heart rate (baseline versus bear presence) among nesting female common eiders (*Somateria mollissima*) on East Bay Island, Nunavut, Canada.

model parameter	variance ± s.d.
sample number [bear event (eider ID)]	2.194 ± 1.481
bear event [eider ID]	22.967 ± 4.792
residual	29.277 ± 5.411

Eider ΔHR ranged from −9 beats/10 s to +40 beats/10 s (mean + 5.08 beats/10 s). Distances from focal eider nests to bears ranged from 12.00 to 286.67 m (mean ± s.d.: 110.81 ± 59.08 m) and the duration of exposure to bears ranged from 1.00 to 14.00 min (mean ± s.d.: 5.67 ± 5.01 min). Size of eider viewshed ranged from 5730 to 33 231 m^2^ (mean ± s.d.: 21 807 ± 9833 m^2^). Incubation stage ranged from 4 to 19 days (mean ± s.d.: 11 ± 5 days). Air temperature ranged from 1.10 to 9.00°C (mean ± s.d.: 3.53̊C ± 2.25°C), and wind speed ranged from 0.00 to 8.30 m s^−1^ (mean ± s.d.: 4.53 ± 2.10 m s^−1^).

**Figure 3.**
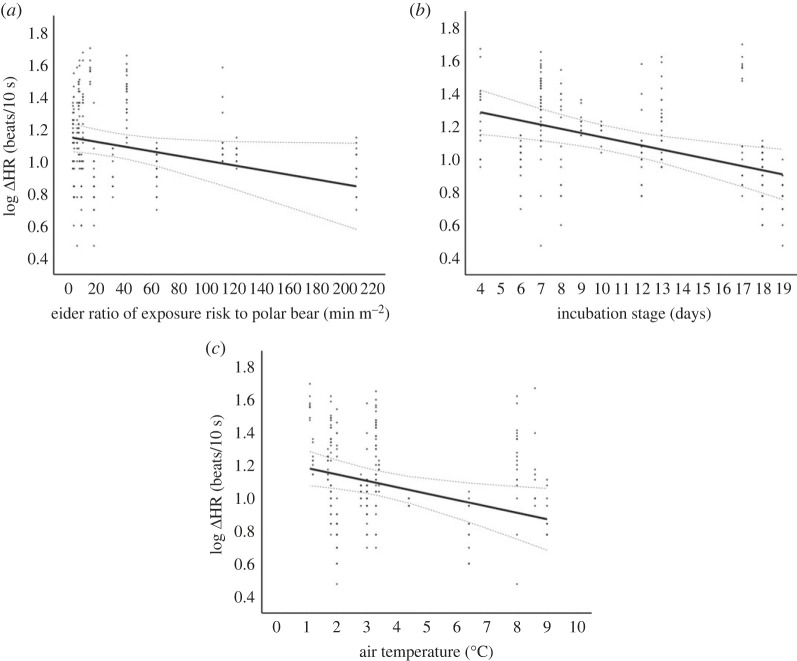
Magnitude of change in log transformed common eider (*Somateria mollissima*) heart rate (‘ΔHR’, beats/10 s) on East Bay Island, Nunavut, Canada in relation to (*a*) eider ratio of exposure risk (min m^−2^) to a polar bear, (*b*) eider incubation stage (days), and (*c*) air temperature (°C). Solid and dashed lines are based on model predicted values ± 95% confidence intervals, respectively.

Our model with log ΔHR as a function of the ratio of exposure risk to polar bears, incubation stage and air temperature best explained our data according to our backward elimination approach (table 3); this was also our top model in terms of AICc and AIC model weight (table 4). In this model, log ΔHR was marginally significantly negatively related to bear exposure time when controlling for viewshed area (*F*_1,16.21_ = 3.84, *p* = 0.067, figure 3*a*), and significantly negatively related to both incubation stage (*F*_1,15.71_ = 8.99, *p* = 0.008, figure 3*b*) and air temperature (*F*_1,17.65_ = 6.10, *p* = 0.024, figure 3*c*). Here, we found that changes in logged heart rate over baseline decreased as bears spent more time within the eider's viewshed, incubation stage progressed and as temperature increased. There was no significant relationship between log ΔHR and distance to the polar bear, nor wind speed, and there was also no significant interaction between the distance to the bear and the ratio of exposure risk to polar bears on log ΔHR. Further, within a bear event itself, heart rate did not differ over the time the eider was exposed to the bear (i.e. across consecutive intervals an eider was exposed to a bear; *F*_35,220.72_ = 0.99, *p* = 0.50).

**Table 3.** Model parameter estimates and standard error for fixed effects used to explain variation in magnitude of change in heart rate among nesting female common eiders (*Somateria mollissima*) in response to polar bears (*Ursus maritimus*) on East Bay Island, Nunavut, Canada.

model parameter	estimate ± s.e.
intercept	1.589 ± 0.139
ratio of exposure risk	−0.001 ± 0.001
incubation stage	−0.025 ± 0.008
air temperature	−0.039 ± 0.016

**Table 4.** Predicting the magnitude of change in heart rate (i.e. ‘ΔHR’, beats/10 s) in female incubating female common eiders (*Somateria mollissima*) in response to polar bears (*Ursus maritimus*) based on AICc model selection (ML estimation). Models included here were used in a backward stepwise regression until significant effects remained (via ML estimation). The null model is represented by ‘ΔHR ∼ 1’.

model	d.f.	logLik	AIC_c_	ΔAIC_c_	*w_i_*
ΔHR ∼ ratio of exposure risk + incubation stage + air temperature	7	84.281	−154.1	0.00	0.42
ΔHR ∼ ratio of exposure risk + incubation stage + air temperature + wind speed	8	85.214	−153.9	0.26	0.36
ΔHR ∼ distance + ratio of exposure risk + incubation stage + air temperature + wind speed	9	85.216	−151.8	2.39	0.13
ΔHR ∼ distance + ratio of exposure risk + incubation stage + air temperature + wind speed + distance × ratio of exposure risk	10	85.645	−150.5	3.68	0.07
ΔHR ∼ 1	4	78.567	−149.0	5.16	0.03

Although, our final model received higher support, according to AICc, than the intercept-only model (table 4), much of the variance explained by our model was accounted for by our random effects (marginal *R*^2^ = 0.25, conditional *R*^2^ = 0.63, table 5). This suggests that variance in eider heart-rate responses is attributable to inter-individual differences, as well as the biotic and abiotic factors we measured. We did not detect collinearity in any fixed effects within each model based on estimated VIFs < 5.

**Table 5.** Model parameter variance and standard deviation for random effects used to explain variation in magnitude of change in heart rate among nesting female common eiders (*Somateria mollissima*) in response to polar bears (*Ursus maritimus*) on East Bay Island, Nunavut, Canada.

model parameter	variance ± s.d.
sample number [bear event (eider ID)]	0.005 ± 0.070
bear event [eider ID]	0.022 ± 0.148
residual	0.026 ± 0.161

## 4. Discussion

Using non-invasive, observational and spatially explicit approaches, we assessed the capacity of common eiders to perceive changes in the degree of threat posed by polar bear predators that eiders are increasingly encountering within their breeding colonies [24,25]. Results from our study show mild support for dynamic risk assessment by incubating eiders to the threat of polar bears. Eiders in the current study exhibited different heart rates in the absence and presence of a bear, and birds also modulated their heart rate to varying levels of predation risk based on the ratio of exposure risk (i.e. exposure duration corrected for viewshed area), but not to varying distance from foraging bears. Results suggest only a minor perceived risk of bears as adult and egg predators. Eiders are a long-lived species [59] and are thus unlikely to have adapted to polar bears by way of rapid evolution [5]. Instead, eiders may have learned to recognize polar bears as a risk to such a degree (though not their full risk) through experience with bears over ontogenetic time (e.g. [60]). Below we discuss the fitness effects eiders are expected to endure based on our results, considering possible inter- and intra-eider sources of variation in heart rate responses to bears.

### 4.1. Heart rate responses to various distances to polar bears

An increased responsiveness to predator proximity has been reported in several nesting bird species (e.g. [7]). While eiders responded to bear presence by increasing heart rate, eiders did not exhibit a noticeable change in heart rate response over baseline with close-proximity bears. Bears in the current study were observed at distances from eiders (i.e. 12–18.5 m) within the range eiders have been reported flushing to disturbance (e.g. 0–20 m to human approaches, [61]; 3–15 m and 3–18 m to approaching Arctic fox and control models, respectively [32]; 0.91–25.7 m to foraging polar bears [8]), but also above the range at which eiders have been reported to flush from visual polar bear stimuli (i.e. 1–9 m [32]). Because neurophysiological responses typically precede any behavioural responses [16,21,62], we would expect that eiders that were preparing for a fight-or-flight response would exhibit an increase in heart rate when bears were closer to their nests, a response commonly associated with the mobilization of energy for quick flight [13]. Thus, our finding that eiders did not vary heart rate responses across varying distance from bears suggest eiders are not responding accordingly and perhaps do not perceive the full risk posed by polar bears.

### 4.2. Heart rate responses to various exposure durations and ratios of exposure risk to polar bears

From the bear's perspective, it could technically see an eider from any location within the eider's viewshed [15]. Therefore, the longer a bear spends within an eider's field of view, the greater chance of the bear detecting and targeting the eider and its nest. Despite this, eiders did not adjust their heart rate the longer that they were exposed to a polar bear during a single event. This lack of response is unlike other examples of heart rate responses to threat in the literature. For example, bystander king penguins (*Aptenodytes patagonicus*) displayed heightened heart rates (i.e. sensitization) the longer the duration of a nearby agnostic encounter since longer conflicts can increase risk of redirected aggression for bystanders [63]. This unchanging heart rate also implies eiders did not dampen their heart rate response (i.e. habituate) over the course of a bear event to reduce the detrimental effects of maintaining high physiological stress responsiveness [64].

Eiders did, however, respond to exposure duration to bears in the context of their visibility (i.e. per unit of viewshed area). Eiders displayed a more reduced heart rate from baseline when their exposures to polar bears were characterized by a larger ratio of exposure duration to visibility risk (max. = exposed to a bear for a long duration in a small viewshed, min. = exposed to a bear for a short duration in a large viewshed). This heart rate response is suggestive of fear bradycardia commonly observed in nesting bird species that use concealment as a predator-avoidance strategy [65] and is therefore not a surprising response given the cryptic camouflage and nesting strategies of female eiders. In addition to exposure duration, an eider's ‘area of visibility’ can be considered a risk factor since eiders with a narrower, and therefore limited, spatial visibility range should perceive a bear detected within their viewshed as a greater risk than an eider with a larger viewshed where risk is ‘diluted’. Additionally, incubating birds are known to compensate for information deficits upon detection of a threat cue by behavioural risk assessment mechanisms such as increasing vigilance (e.g. incubating brown thornbill *Acanthiza pusilla* to predator calls [20]); and heart rate responses have been positively linked to vigilance following the detection of a threat in several species of birds [17]. Thus, the regulation of heart rate in relation to variation in large and small ratio of exposure risk suggests female eiders dynamically updated their assessment of risk to bears and thus perceived polar bears as a threat using this predator cue.

### 4.3. Other possible indicators of risk of polar bear predation

A weak or lack of signal of predator perception may be attributable to individual characteristics of the bears [18]. To assess risk, eiders must use a complex set of overt and subtle predatory cues that provide information on a predator's likely future actions. Direct and rapid predator approaches, and those where the predator's head and gaze directly face the focal prey, are presumably perceived as a greater risk than tangential and slower approaches or when the predator's head and gaze are averted [9,10]. In the current study, we were unable to quantify possible predator cues such as whether bears were mobile, their speed and their direction of attention (i.e. body, head and gaze) in relation to focal eiders, which may explain the lack of response to our ‘bear-distance’ measure. Recent work by Barnas *et al*. [8] has been conducted to assess flushing responses of eiders to multiple subtle cues by foraging polar bears.

### 4.4. Intra- and inter-individual variation in physiological responses

We found intra- and inter-individual variation in the magnitude of change in heart rate (from baseline heart rate), which was accounted for by variation in incubation investment and abiotic meteorological conditions. Specifically, the heart rate responses of eiders declined over the incubation period. Perhaps heart rate responses associated with flight are reduced as females invest more into reproduction compared with survival (e.g. [66]), or in attempts to save energy as they become more energetically limited while fasting [37]. This dampening of their stress response as incubation progresses is consistent with predictions of nest defence theory, and follows similar patterns to other waterfowl species responding to threat (e.g. human disturbance) [67]. Heart rate responses also increased with declining temperature. Endothermic animals typically increase metabolic heat production to regulate their body temperature in low ambient temperatures, which requires an increase in cardiac output and thus heart rate [68,69]. Gabrielsen *et al*. [70] studied eider thermoregulation in a field-laboratory setting and found that incubating eider metabolism increased as ambient temperature decreased below eiders' lower critical temperature of 7°C. Indeed, in most cases, eiders in the present study were exposed to temperatures below this lower critical temperature which explains increasing heart rates with decreasing temperature. However, our absence of influence of wind speed on eider heart rate is surprising since wind can dissipate body heat by disrupting plumage and reducing thermal insulation of feathers [62]. It is possible postural changes, where birds may be altering their overall height on the nest to reduce exposure to wind, and/or the use of plumage compression to avoid deleterious cooling effects of the wind [71] could both result in decreased energetic expenditure and hence unaltered heart rate responses.

## 5. Conclusion

Our findings provide new insights on the adaptive capacity for eiders to perceive variation in risk posed by their increasingly frequent interaction with polar bear egg predators. We assume a fitness-driven role for dynamic risk assessment in birds since heart rate responses can affect individual fitness via its associated energetic costs (e.g. [72]). Our finding that eiders increased their heart rate when in the presence of polar bears suggests eiders are able to recognize polar bears as threatening, but are unable to attenuate this response accordingly over varying durations of a bear event, suggesting eiders are not displaying ‘optimal’ physiological responses to bears. Eiders, do, however, update their responses to one of the conspicuous indicators of risk (ratio of exposure risk). Nonetheless, eider hens' lack of a measurable physiological response to distance to bear suggests eiders do not recognize the full risk that bears pose to adult and nest predation. As such, eiders may lack the capacity to mitigate the increased frequency of predator interactions (i.e. modifying flush distances, distraction displays, etc.) to reduce predation, and are thus expected to endure negative reproductive fitness effects from the increasing frequency of interactions with polar bears [6]. The effect of polar bear predation on the reproductive fitness value of hens can be dramatic [24], so fluctuations in eider population size may now be driven by more external factors (e.g. polar bear nest predation) beyond the traditional drivers of annual productivity (e.g. feeding conditions) [73].

## Data Availability

Data for our ‘Statistical Analyses' section of our manuscript can be accessed in the ‘Eider ∆HR Response.csv’ and ‘Heart Rate Type.csv’ files in electronic supplementary material. Code for our ‘Statistical Analyses' section of our manuscript can be accessed in the ‘R Code.txt’ file in electronic supplementary material. Netlogo code can be accessed in the ‘Netlogo Code.txt’ file in electronic supplementary material. Supporting GIS files for these simulations can be found in ‘Camera Locations.csv’ and ‘GIS Files.zip’ files in electronic supplementary material. All parameter values have been identified in the electronic supplementary material. The data are provided in the electronic supplementary material [74].
